# Operation of Droplet-Microfluidic Devices with a Lab Centrifuge

**DOI:** 10.3390/mi7090161

**Published:** 2016-09-06

**Authors:** Noorsher Ahmed, David Sukovich, Adam R. Abate

**Affiliations:** Department of Bioengineering and Therapeutic Sciences, California Institute of Quantitative Biosciences (QB3), University of California, San Francisco, CA 94115, USA; noorsher2@gmail.com (N.A.); d.sukovich@gmail.com (D.S.)

**Keywords:** microfluidics, droplets, poly(dimethylsiloxane), lab on chip

## Abstract

Microfluidic devices are valuable for a variety of biotechnology applications, such as synthesizing biochemical libraries, screening enzymes, and analyzing single cells. However, normally, the devices are controlled using specialized pumps, which require expert knowledge to operate. Here, we demonstrate operation of poly(dimethylsiloxane) devices without pumps. We build a scaffold that holds the device and reagents to be infused in a format that can be inserted into a 50 mL falcon tube and spun in a common lab centrifuge. By controlling the device design and centrifuge spin speed, we infuse the reagents at controlled flow rates. We demonstrate the encapsulation and culture of clonal colonies of red and green *Escherichia coli* in droplets seeded from single cells.

## 1. Introduction

Droplet microfluidic techniques [1,2,3] are valuable for a variety of applications in biology, including for evolving enzymes with enhanced function [4,5] and analyzing huge numbers of single cells at ultrahigh throughput [6,7,8]. The most common microfluidic devices are fabricated in the elastomer poly(dimethylsiloxane) (PDMS) and operated using variable-control syringe pumps [9,10]. Syringe pumps allow precision control and variation of flow rates to optimize device operation; however, they are expensive, imposing a barrier to the adoption of droplet microfluidics by non-experts. Moreover, once a particular microfluidic design has been optimized, the same flow rates are generally used to operate it, obviating the need for variable control pumps. In such instances, passive pumping with pressure [11], suction [12,13], or gravity-driven flow [14] is preferable, since it demands less of the user and makes adoption easier.

An especially effective method for pumping fluids through devices is with centrifugation, a technique known as centrifugal microfluidics [15,16]. These devices are commonly fabricated in a compact disc format with channels etched between two plastic plates. Reagents are loaded into the device and infused by spinning the disc. This approach is simple and robust in the hands of non-experts [17,18]. Nevertheless, the fabrication methods to construct the polycarbonate disc devices are non-trivial, requiring expertise with injection molding or embossing [19,20,21]. Soft-lithography in PDMS is far more common, and is a robust and well-understood process. It is easy to get started with and can be used for rapid prototyping or mass production. An ideal system for operating microfluidic devices would allow the use of common PDMS devices with a lab centrifuge.

In this paper, we demonstrate operation of PDMS devices using a common lab centrifuge. We build a scaffold, which can hold a device and reagents to be infused, small enough to be inserted into a 50 mL falcon tube, allowing the assembly to be spun with a common lab centrifuge. By controlling device dimensions, reagent reservoir heights, and spin speed, we generate controlled pressure drops that inject multiple reagent streams at different, specified flow rates. We demonstrate how to control flow rates in a cross-flow droplet generator and use the approach to encapsulate and culture different fluorescent strains of *Escherichia coli* (*E. coli*) seeded from single cells. Our approach is compatible with any assay that uses a droplet maker. With additional inlet ports, it can be extended to more complex devices, such as co-flow droplet makers that allow the merger of distinct aqueous streams immediately prior to droplet generation [22,23,24,25] or the synthesis of multiple emulsions [26,27,28]. It provides a simple and general means by which to operate PDMS devices with a common lab centrifuge.

## 2. Materials and Methods

**Figure 1:**
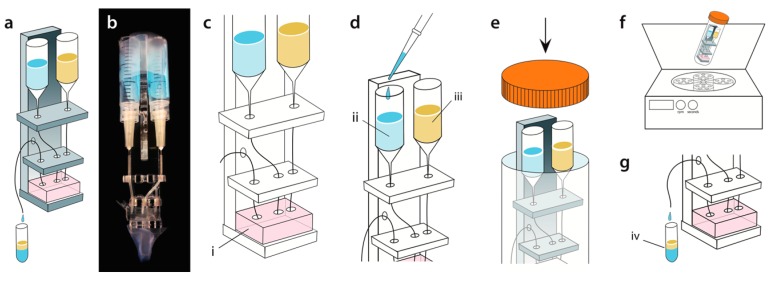
Schematic and workflow of centrifuge droplet generation. (**a**) Illustration of microfluidic cross-flow PDMS droplet generator with 25 µm by 25 µm nozzle dimensions on a plastic device holder; (**b**) A photograph of the actual plastic holder with the PDMS droplet generator, syringe reservoirs, tubing, and collection tube fully installed; (**c**) The PDMS droplet generator (i) is installed on the plastic holder; (**d**) A pipette tip is used to load the aqueous (ii) and oil (iii) into the syringe reservoir; (**e**) To generate droplets, the device is loaded into a standard 50 mL tube and spun in a centrifuge; (**f**,**g**) During centrifugation, hydrostatic pressure generated by spinning pumps the fluids through the droplet generator and produces the emulsion (iv).

We fabricate the flow-focusing microfluidic droplet generators from PDMS using the techniques of soft lithography [29]. PDMS replicates of SU8 molds are cut from the wafer and interfaced with access ports via a 0.75 μm biopsy punch. The devices are plasma-bonded to a PDMS slab and treated with Aquapel to render them hydrophobic [30]. The droplet generator’s cross-flow junction is 25 µm by 25 µm, and the fluidic resistance channels for both inlets are 20.5 mm long, with cross-sectional dimensions 25 µm wide by 50 µm tall. The centrifugation scaffold is designed in AutoCAD and assembled from 3.175-mm-thick acrylic plastic sheets prepared with a ULS Laser Cutter (Figure 1a,b, Universal Laser Systems, Scottsdale, AZ, USA). The parts are bonded with acrylic cement (Tap Plastics, Oakland, CA, USA). Reservoirs for the sample and carrier fluids are prepared by sawing 3 mL plastic syringes (BD) in half before attaching the needles (BD Precision Glide, Becton, Dickinson and Company, Franklin, NJ, USA) and polyethylene tubing (Scientific Commodities, Lake Havasu City, AZ, USA) (Figure 1b).

The reservoirs are positioned such that the 0 mL mark of the syringe is about 50 mm above the microfluidic device (Figure 1c). The device is primed with oil in all reservoirs, removing air bubbles that may alter fluidic resistances. The tubing through which reagents are introduced are also filled with HFE-7500 oil via a syringe and cut to the length needed for the appropriate reservoir height. The cut-end is then inserted into the inlet of the device, and the other connected to the reagent reservoir via its needle, being careful to not trap air in the tubing. The reservoir is mounted on the scaffold and affixed with tape (Figure 1d). The generated droplets are collected into a polymerase chain reaction (PCR) tube taped to the bottom of the scaffold (Figure 1a,b, lower). The assembly is loaded into a 50 mL falcon tube for centrifugation (Figure 1e,f). The centrifuge is set to a speed and time to obtain droplets with the desired diameter and volume of total emulsion. After centrifugation, the collection tube containing the droplets is recovered (Figure 1g).

Even though we typically use <1 mL of reagent per run, we use 3 mL syringes for the fluid reservoirs because they are wide; as fluid is drawn from the reservoirs, the height of the top of the fluid with respect to the device changes negligibly, which maintains a constant flow rate. For example, for 500 μL of aqueous sample with a density of 1 g/cm^3^ at a height of 50 mm, producing 100 μL of drops yields a change in reservoir height of ~0.4 mm which, for 20 g and a representative hydrodynamic resistance of 124 kPa·h/µL, results in a variation in flow rate of ~1%. This is a tighter tolerance than is commonly achieved using syringe pumps, which can fluctuate in flow rate by over 10% due to the stepper motor [31].

We use phosphate buffered saline as the aqueous fraction and 3M™ Novec™ 7500 Engineered Fluid containing 2% ionic Krytox as the oil fraction in the test emulsions. Reagents are loaded into the inlet reservoirs, and droplet makers spun at speeds ranging from 200 to 950 rpm, to identify ideal flow rates for the making of monodisperse droplets. The emulsions are visualized using an inverted microscope in bright field mode. The centrifuge-made droplets are generated at 500 rpm achieving flow rates of 200 µL/h and 300 µL/h for oil and aqueous phases, respectively. The pump-made emulsions are generated with the same flow rates.

For experiments utilizing *E. coli* expressing green fluorescent protein (GFP) or red fluorescent protein (GFP cloned into pET-28a in BL21(DE3) and mCherry cloned into pET-28a in BL21(DE3) respectively), *E. coli* strains are grown overnight in magic media (Invitrogen) supplemented with kanamycin. Cells are then diluted to 1.99 × 10^6^ cells/mL to achieve approximately one cell per 10 droplets in a mixture of magic media supplemented with optiprep (20%) to prevent sedimentation of the cells during infusion of the device. Droplets of the sample are generated in a centrifuge at 500 rpm for 10 min. After emulsification using HFE supplemented with 2% PEG-Krytox surfactant, samples are allowed to grow overnight at 37 °C and visualized using a fluorescent microscope.

## 3. Results

To obtain a controlled flow rate through a channel, a pressure drop must be applied. This can be achieved by pressurizing the inlets with syringe pumps or pressure regulators, or by applying vacuum to the outlet [13]. Our strategy is to use a centrifuge. Since the reagent reservoirs are above the channels, the hydrostatic pressure of the fluid at the inlet is higher than at the outlet. Moreover, the centrifuge allows us to vary hydrostatic pressure by spinning at different angular rotation velocities, in accordance with

(1) P= ρ(ag)s

where ρ is the fluid density, a is a constant that can be adjusted with the centrifuge, g is the Earth’s gravitational constant of 9.81 m/s^2^, and s is the distance of the fluid in the syringe reservoir from the microfluidic device. For a given pressure drop ΔP, the flow rate (Q) through the channel depends on its hydrodynamic resistance ($R_{hyd}$),

(2) $$Q=\frac{\Delta P}{R_{hyd}}$$

where the resistance for a channel with a rectangular cross-section can be approximated as

(3) $$R_{hyd}=\frac{12}{\left(1-\left(\frac{192}{\pi^{5}}\right)\left(\frac{h}{w}\right)\left(\tanh\left(\frac{\pi }{2}wh\right)\right)\right)}\left(M\frac{L}{wh^{3}}\right)$$

where w is the width of the channel, h is the height, L is the length, and M is the viscosity of the fluid. When aqueous and oil phases are injected into a hydrophobic droplet generator of fixed dimensions, the resultant droplet volume depends on the ratio of the flow rates of the phases,

(4) $$\frac{l}{w}=1+\alpha \frac{Q_{aq}}{Q_{total}}$$

where l is the length of the drop formed, w is the width of the channel, α is a constant that depends on the channel geometry and is close to 1 [32,33,34]. Hence, by varying the centrifugation speed, reservoir height, and inlet resistance, we can control the relative and absolute flow rates of the phases, thereby generating droplets of controlled size.

**Figure 2:**
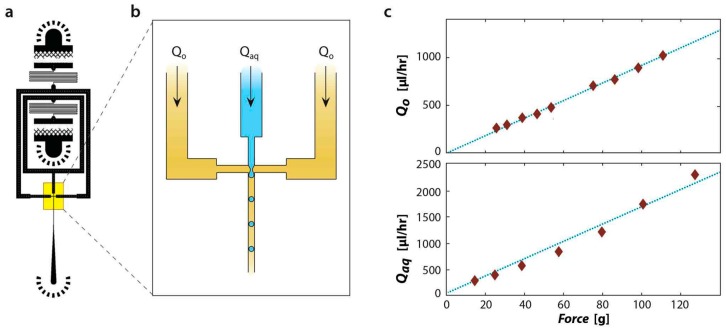
Centrifugation allows controlled flow of aqueous and oil through a microfluidic droplet generator. (**a**) CAD schematic of device and (**b**) magnified view of droplet generation region. The droplet generator nozzle is 25 μm wide by 25 μm tall; (**c**) Measurement of oil and aqueous flow rates as a function of centrifugation force.

As when using syringe pumps to drive fluids through a microfluidic device, our method allows different fluids to be introduced at different, controlled flow rates. To illustrate this, we measure the flow rates of aqueous and oil phases through a PDMS device outfitted with fluidic resistance channels (switchbacks, Figure 2a). The fluidic resistance channels are designed to have ~50-fold higher resistance than the rest of the device, ensuring that a majority of the pressure drop is through the resistance channels and, hence, that flow rates depend primarily on their dimensions for a given applied pressure drop. The droplets form in a flow focus junction by infusing water and oil from different channels (Figure 2b). To measure flow rates through the different paths, we plug one inlet while flowing liquid through the other, and vary the centrifugation speed. We collect the fluid exiting the device after a controlled time and measure its mass, the results of which are plotted in Figure 2c for the oil and aqueous phases, respectively. We find that the flow rates are, as expected, directly proportional to the centrifugation speed. The experiment was reproduced three times, each time showing a definitive linear relationship between the flow rate and centrifugation speed.

**Figure 3:**
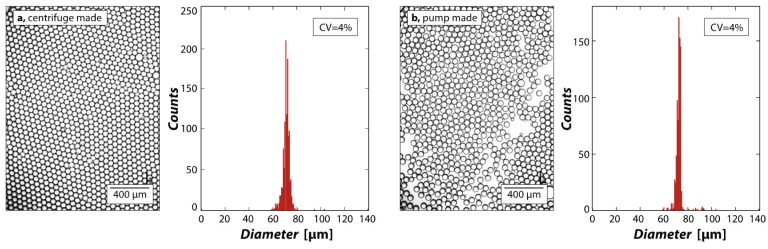
Centrifugal pumping generates droplets of equivalent monodispersity to syringe pumps. (**a**) Droplets generated with a flow-focus device using a centrifuge are monodisperse, with an average diameter of 71 µm and standard deviation of 2 µm; (**b**) Droplets made using a syringe pump are equally monodisperse, with an average diameter of 72 µm and standard deviation of 2 µm.

For the device to be valuable for performing biological assays, it must form droplets of uniform size. To investigate this, we create droplets with a PDMS flow-focusing droplet generator and image samples of the resultant droplets, shown in Figure 3a. The centrifuge-made droplets are monodisperse, as exemplified by their hexagonal packing and confirmed by their narrow size distribution, having a coefficient of variation of ~4%, as shown in Figure 3a, left. To compare these results with standard pump-made emulsions, we use the same device to generate an emulsion using syringe pumps, as shown in Figure 3b. We again achieve high monodispersity and a coefficient of variation of ~4%, as shown in the figure. This validates that the centrifugation-based operation of a droplet generator provides uniformity of operation that is similar to pump-based operation. Furthermore, the achieved monodispersity is comparable to other techniques, such as polyester toner devices [35].

**Figure 4:**
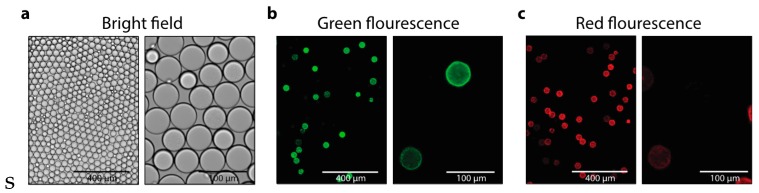
GFP- and RFP-expressing *E. coli* co-encapsulated and cultured in emulsions (left column = 10× magnification; right column = 40× magnification). The emulsions are visualized after the cells have had time to grow with bright field microscopy (**a**); and fluorescence microscopy with a GFP-filter (**b**); and RFP-filter (**c**).

To validate the efficacy of centrifuge-made emulsions for biological assays, we use them to encapsulate and culture fluorescent cells. The sample fluid consists of a mixture of two *E. coli* strains expressing green fluorescent protein (GFP) and red fluorescent protein (RFP), respectively. We partition the sample such that most droplets are empty and a small fraction contains single cells, in accordance with Poisson statistics

(5) $$P\left(x;\lambda \right)=\frac{e^{-\lambda }\lambda^{x}}{x!}$$

where x is the number of cells in a droplet and λ is the average number of cells per droplet [36,37,38,39]. Spinning the sample can cause the cells to sediment to the bottom of the inlet reservoirs. However, we density-match the cells using optiprep, to slow sedimentation. Additionally, for bacteria of this size, 5000 rpms for 10 min is commonly used for the concentration, while we spin our device only at 500 rpms for 10 min, such that sedimentation is negligible. The emulsion is collected and incubated, allowing the cells to divide, and imaged with a fluorescence microscope. As expected, we find that most droplets are empty with a small fraction containing fluorescent cells. Since the encapsulation of the two cell types is random, droplets containing cells are most often GFP- or RFP-positive, with a small fraction double positive, such as the droplet depicted on the upper-left of the images in the second column of Figure 4b,c. Droplets containing cells tend to shrink relative to empty droplets, due to cells consuming media and generating osmotic pressure differences that are equalized by droplet shrinkage [37]. This demonstrates that our approach can be used to encapsulate and culture cells in droplets without the use of pumps. Since this is a first step in most single-cell analysis applications, such as the analysis of secreted compounds or the sequencing of single-cell transcriptomes via droplet barcoding [40,41], this demonstrates that our approach can be used for these valuable applications.

## 4. Conclusions

We have developed an approach for pumping fluids through microfluidic devices using a standard lab centrifuge. Our approach is applicable to common PDMS devices and allows multiple reagent streams to be infused at controlled flow rates by properly setting the fluid reservoir height, inlet channel resistance, and centrifuge speed. The method consists of a scaffold on which different microfluidic chips can be mounted; the number of inlets can be increased by adding more fluid reservoirs to infuse additional fluids and operate more complex devices. The ease of our approach, combined with its reliance on a common lab centrifuge, makes it easier to adopt than methods that require pumps or mastery of complex fabrication techniques. We have demonstrated the use of the approach for performing cell encapsulation and culture assays and anticipate that it will be useful for other “digital’’ assays, such as culture-based screens, digital PCR, and digital ELISA. By incorporating wettability-patterning, it should also be useful for facile formation of monodisperse double emulsions and lipid vesicles.
